# Hepatitis B virus–host interactions and novel targets for viral cure

**DOI:** 10.1016/j.coviro.2021.04.009

**Published:** 2021-05-22

**Authors:** Gaëtan Ligat, Eloi R Verrier, Michael Nassal, Thomas F Baumert

**Affiliations:** 1 Université de Strasbourg, F-67000 Strasbourg, France; 2 Inserm, Institut de Recherche sur les Maladies Virales et Hépatiques UMRS 1110, F-67000 Strasbourg, France; 3 University Hospital Freiburg, Dept. of Internal Medicine 2/Molecular Biology, D79106 Freiburg, Germany; 4 Institut Hospitalo-Universitaire, Pôle Hépato-digestif, Nouvel Hôpital Civil, 67000 Strasbourg, France

## Abstract

Chronic infection with HBV is a major cause of advanced liver disease and hepatocellular carcinoma. Nucleos(t)ide analogues effectively control HBV replication but viral cure is rare. Hence treatment has often to be administered for an indefinite duration, increasing the risk for selection of drug resistant virus variants. PEG-interferon-α-based therapies can sometimes cure infection but suffer from a low response rate and severe side-effects. CHB is characterized by the persistence of a nuclear covalently closed circular DNA (cccDNA), which is not targeted by approved drugs. Targeting host factors which contribute to the viral life cycle provides new opportunities for the development of innovative therapeutic strategies aiming at HBV cure. An improved understanding of the host immune system has resulted in new potentially curative candidate approaches. Here, we review the recent advances in understanding HBV–host interactions and highlight how this knowledge contributes to exploiting host-targeting strategies for a viral cure.

## Introduction

Chronic hepatitis B (CHB), caused by hepatitis B virus (HBV), is a major cause of advanced liver disease and hepatocellular carcinoma (HCC), the second leading cause of cancer death worldwide [1•,2••]. HBV remains a major public health concern with an estimated global prevalence of 250–300 million chronic virus carriers [3].

Although available drugs against CHB including PEG-interferon-α-based therapies and nucleos(t)ide analogues (NUCs) can effectively control HBV replication, viral cure is extremely rare, for NUCs even upon decade-long treatment [4]. NUCs including lamivudine, adefovir, tenofovir or entecavir directly inhibit the reverse transcriptase activity of the HBV polymerase. Hence resistance mutations occurring during long-term treatment usually map to the reverse transcriptase (RT) domain of the polymerase [5,6] and its individual subdomains. Resistance to lamivudine is conferred by mutations within the catalytic C domain (YMDD motif), resistance to entecavir by mutations in the B, C or D domains. Initial mutation-induced reductions in enzymatic efficiency may be compensated by additional mutations which enhance viral replication [6–8].

CHB is characterized by the persistence of the episomal covalently closed circular DNA (cccDNA) form of the HBV genome which persists as a stable minichromosome in the nuclei of infected hepatocytes [9]. After therapy withdrawal or loss of immune control, a few cccDNA copies per hepatocytes can reactivate full viral replication. Hence cccDNA would have to be eliminated from infected liver cells to achieve HBV cure. Such sterilizing cure, that is, complete viral eradication from the host, is the ultimate but at present hardly achievable goal. A more feasible objective is ‘functional cure’, with seroclearance of hepatitis B surface antigen (HBsAg) as a defining parameter [10••]. For either type of CHB cure the development of new therapeutic strategies remains a key unmet medical need [10••].

Targeting host factors involved in the viral life cycle is a promising and increasingly successful therapeutic approach to overcome resistance by new inhibitors that are characterized by a high genetic barrier. Several host targeting agents (HTA) have already been transferred into clinic. Notably, Maraviroc is a specific antagonist of the human immunodeficiency virus-1 (HIV-1)CCR5 chemokine receptor. It inhibits HIV-1 from entering host cells and is clinically approved for anti-HIV treatments [11]. Furthermore, Bulevirtide (formerly known as Myrcludex B), a peptide specifically targeting the HBV and hepatitis D virus (HDV) entry factor sodiumtaurocholate cotransporting polypeptide (NTCP), has been approved in the European Union in 2020.

Developing new HTA to control and/or possibly cure viral infection requires a comprehensive characterization of virus-host molecular interactions, including targetable cell factors playing key roles in the viral life cycle. The recent development of high-throughput functional genomics applied to virus infection systems paved the way for the identification and characterization of many such factors. Numerous genome-scale loss-of-function screens via RNAi knockdown and CRISPR/Cas9 knock-out have been successfully applied to hepatitis viruses, HIV-1, influenza A and Zika virus [12•,^13^,14•, 15–17]. For instance, EGFR was found necessary for hepatitis C virus (HCV) entry [18]; an siRNA screen identified CD97, NEIL3, BMP2K, and SERPINB6 as HIV-1 host factors and antiviral targets [15]; and a genome-wide CRISPR screen defined WDR7,CCDC115 and TMEM199 as crucial host dependency factors for influenza A virus infection[16]. Complementary to such loss-of-function studies, a genome-wide gain-of-function screen recently identified the cyclin-dependent kinase inhibitor CDKN2C as a key host factor for HBV [14•].

In this review, we present the cellular functions known to be involved in key steps of the HBV life cycle, discuss the therapeutic potential of individual HBV-related host factors, and highlight the molecules currently in development for novel therapeutic strategies that may lead to HBV cure.

## Molecular virology of HBV infection

HBV is an enveloped hepatotropic DNA virus belonging to the *Hepadnaviridae* family, characterized by infectious virions carrying a 3.2 kb relaxed circular DNA (rcDNA) genome which is produced by reverse transcription. Viral entry initiates with the attachment to heparan sulfate proteoglycans including glypican 5 (GPC5) [12•] and is followed by a specific binding to a high-affinity virus receptor, the bile acid transporter NTCP [19••,^20^]. Internalization of the NTCP-HBV complex via clathrin-dependent endocytosis [21] is mediated by the EGF receptor (EGFR) [22]. How the envelope is stripped off is poorly understood. However, once in the cytoplasm the viral capsid is transported, via interactions of nuclear localization signals in the capsid protein with importins, along microtubules [23] to the nuclear pore complex (NPC), where the viral rcDNA is released into the nucleus and converted into cccDNA. As a result of the unusual protein-primed reverse transcription mechanism [24,25], recently shown to be an ancient principle of circular genome replication [26], rcDNA carries several molecular peculiarities that must be repaired for cccDNA formation. The 5^′^ end of the minus-strand in rcDNA is covalently linked to the viral polymerase [27] and carries a short terminal redundancy; the plus-strand starts with a capped RNA oligomer and has heterogeneous, incomplete 3^′^ ends. While the details of rcDNA to cccDNA conversion are far from being understood it is highly plausible that HBV usurps host DNA repair factors to remove the non-DNA moieties and surplus sequences, and to fill-in and eventually ligate the gaps to generate cccDNA as a proper template for viral transcription. As each nt in the hepadnaviral genome has coding function in one or even two open reading frames all the repair reactions must proceed with single nt accuracy [28••].

Meanwhile various DNA repair associated factors have indeed been identified as hepadnaviral host dependency factors. Using duck HBV (DHBV) in human hepatoma cells as a high-copy cccDNA model for HBV, Königer and collaborators found Tyrosyl-DNA-phosphodiesterase 2 (TDP2) as capable of releasing the viral polymerase from rcDNA [29]. DNA polymerase K (POLK) provides an important DNA fill-in activity during cccDNA generation [30], and DNA ligases 1 and 3, but not DNA ligase 4, are the key host ligases in cccDNA strand closure [31]. The cellular ATR-CHK1 DNA damage repair pathway was shown to be involved in rcDNA processing and HBV cccDNA formation [32]. More detailed characterization of conversion intermediates is helping to further decipher the pathway, or possibly redundant pathways, from rcDNA to cccDNA [33]. Very recently, five core components of cellular lagging-strand DNA synthesis were identified as the minimal set of factors necessary for *in vitro* rcDNA to cccDNA conversion: proliferating cell nuclear antigen, the replication factor C complex, DNA polymerase δ, flap endonuclease 1 and DNA ligase 1 [34•], and their strand-specific contributions have been defined [35]. Likely these factors are also relevant for cccDNA formation in vivo yet the redundancy in host DNA repair factors and the need for intracellular genome transport imply that in cells many additional factors contribute to cccDNA biogenesis and regulation [28••]; these may be easier to target therapeutically than the basic cellular DNA replication machinery.

**Figure 1:**
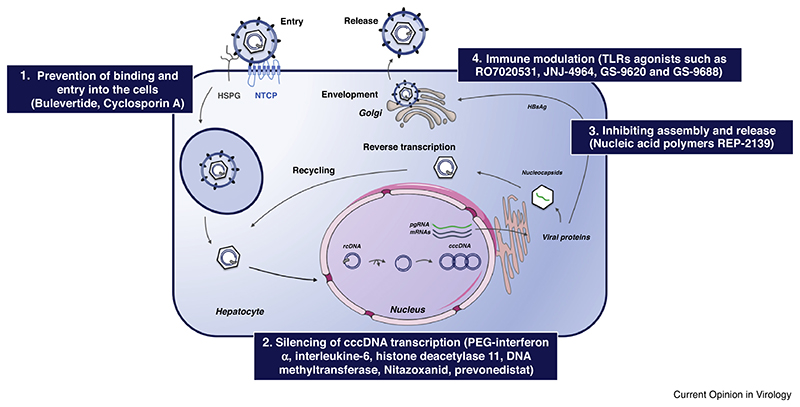
Host-dependency factors of the HBV life cycle as antiviral targets (adapted from Ref. [4]). Following binding of the virus to glypican 5 and HSPG, NTCP as high affinity receptor mediated HBV entry into the hepatocytes. Following cell entry, the nucleocapsid transports the rcDNA to the nucleus. There rcDNA is converted into an episomal cccDNA minichromosome that serves as a template for all viral transcripts including pgRNA which is encapsidated and reverse transcribed into new rcDNA. The nucleocapsids can be enveloped and released as virions or be recycled to the nucleus to replenish the cccDNA pool. There remains an unmet need to develop new therapeutic strategies aiming to overcome resistance. Every step of the HBV life cycle is dependent on the host factors, which can be explored as antiviral targets for the development of new therapeutic approaches. Examples for host-targeting strategies: (1) Targeting host-dependency factor of the HBV entry and binding. (2) Silencing of cccDNA transcription by host epigenetic factors. (3) Targeting host factors required in the last steps of the HBV life cycle. (4) Immune modulation. Abbreviations: relaxed circular DNA (rcDNA), covalently closed circular DNA (cccDNA), pregenomic RNA (pgRNA), sodium taurocholate cotransporting polypeptide (NTCP), heparan sulfate proteoGlycan (HSPG).

In vivo, cccDNA associates with cellular histones and nonhistone-proteins, and likely also viral proteins, into a minichromosome as the actual transcription template. Similar to host DNA its activity is regulated by epigenetic modifications [36]. From cccDNA RNAs of different lengths are produced which direct the synthesis of viral surface proteins, polymerase, core protein (HBcAg and HBeAg) and HBx. The assembly and transport processes are mediated by host trafficking proteins; host chaperones, such as heat shock protein 70 (Hsp70), Hsp40 and Hsp90 interact with the viral polymerase [37] to mediate capsid-internal reverse transcription of one of the transcripts, the pregenomic (pg) RNA into new rcDNA. Various host factors are reportedly incorporated into the capsid, including eukaryotic translation initiation factor 4E (eIF4E), APOBEC3G and the DEAD-box RNA helicase DDX3 [38–40]. The rcDNA-containing nucleocapsids can either be enveloped by the viral surface proteins and released as viral particles (secretion pathway), or can be recycled and translocated into the nucleus to maintain cccDNA levels (recycling pathway) [41]; likely these decisions are also governed by host factors. In sum, every step of the HBV life cycle is dependent on host factors, which therefore represent a wealth of antiviral targets to be explored for the development of new therapeutic approaches (Figure 1 and Table 1).

## Targeting NTCP for the inhibition of virus entry

As the first step in the viral life cycle entry is a conceptually attractive target for the prevention and, if chronicity involves reinfection, also treatment of virus infection. Targeting HBV entry into hepatocytes was extensively studied following the discovery of NTCP as a receptor for HBV and HDV which hijacks the HBV envelope to produce infectious particles (Figure 1 and Table 1). A large number of NTCP inhibitors have been described to exhibit significant anti-HBV activity *in vitro* (including irbesartan, ezetimibe, and ritonavir) [42]. Cyclosporin A (CsA), a well-known immunosuppressive agent, can directly bind to NTCP and interrupt the interaction between NTCP and the HBV attachment factor, that is, the preS1 region of the large envelope protein, and thus block infection [43,44]. Meanwhile non-immunosuppressive cyclosporine derivatives such as SCY446 and SCY450 have been characterized for their capacities to inhibit HBV entry without affecting the transporter function of NTCP [45]. Already before the identification of NTCP as a HBV receptor, the myristoylated preS1 domain was shown to mediate the specific interaction of HBV with hepatocytes, and fatty acylated peptides derived from preS1, such as Myrcludex B (Bulevirtide) had exerteded remarkable antiviral activity in vivo [46]. The demonstration that such peptides bind specifically to NTCP, block *de novo* HBV infection and suppress intra-hepatic viral spreading, prompted clinical trials [47]. Although of limited efficacy against CHB, Bulevirtide has shown robust activity in controlling, or even curing, chronic hepatitis D infection (CHD) in monotherapy but especially when combined with interferon α (IFN-α) [48]. The entry inhibitor has thus recently been approved in the European Union for the treatment of CHD in patients with compensated liver disease [49••] and may find even more widespread use when now marketed by US-based Gilead Sciences.

## Targeting cccDNA biology

Elimination of HBV cccDNA, the persistence reservoir in infected hepatocytes, is the ultimate approach towards an HBV cure. As this review focuses on HTAs we will only briefly consider direct strategies such as sequence-specific CRISPR/Cas targeting of cccDNA. Though seemingly straightforward, there a various caveat beyond classical off-target effects. Particular attention will have to be paid to chromosomally integrated HBV DNA; recent data suggest that although integration is a nonproductive pathway for the virus it occurs already early after infection [50]. Hence CRISPR/Cas induced double-stranded DNA breaks (DSBs) could jeopardize chromosome integrity; in addition, the intended DSBs in cccDNA would create linear DNAs which are preferrred substrates for new integration events. Using small molecules targeting the cccDNA pool may be another therapeutic strategy (Figure 1 and Table 1) although it is difficult to envisage how specificity for viral as opposed to host DNA would be achieved. Nonetheless, screening a limited diversity small-molecule library two compounds, CCC-0975 and CCC-0346, showed an inhibitory activity on HBV infection by reducing the levels of deproteinized rcDNA and cccDNA, possibly by interfering with rcDNA to cccDNA conversion [51]. In the same vein, hydrolysable tannins (punicalagin, punicalin and geraniin) were reported to reduce cccDNA levels by impairing cccDNA formation and promoting its decay [52]. As cccDNA formation is highly dependent on cellular factors, especially from the DNA repair machinery, targeting these factors may represent another option for eventual cccDNA elimination. Various small molecules have been shown to target DNA repair host factors, including DNA ligases, PARP, ATR, ATM and CHK, suggesting them as anti-cancer strategies [53–55]. The PARP inhibitor olaparib, for instance, is in phase 2 clinical trials for the treatment of various cancers [54]. An experimental study has shown that lymphotoxin-β-receptor and IFN-α activation upregulate expression of apolipoprotein B mRNA-editing enzyme catalytic polypeptide (APOBEC) proteins such as APOBEC 3A and 3B; these cytidine deaminases may cause cccDNA deamination, apurinic/apyrimidinic site formation and eventually reduced cccDNA levels [56••]. Notably in this and other studies about one third of the cccDNA seemed resilient against this reduction; deciphering whether this relates to distinct forms of cccDNA or perhaps distinct classes of cells harboring the cccDNA molecules remains an important issue for successful future therapies.

## Silencing of viral transcription

A less demanding alternative to cccDNA eradication is silencing of its transcriptional activity. As for CRISPR/ Cas we will here not discuss RNAi mediated silencing of HBV transcripts [57] although potentially beneficial indirect effects are beginning to be investigated [58].

As mentioned the transcriptionally active form of cccDNA is a minichromosome that is subject to epigenetic modifications [36] (Figure 1 and Table 1). cccDNA encodes four promoters and two enhancers which feature various transcription factor binding sites, including for HNF1, HNF3, HNF4, the retinoid X receptor (RXR) and the CCAAT-enhancer-binding protein (C/EBP) [59–61]. Hence cccDNA transcription and its regulation are broadly dependent on host factors, opening various options for therapeutic cccDNA transcriptional control [62] (Figure 1 and Table 1). For instance, IFN-α and interleukin-6 (IL-6) reportedly decrease cccDNA-bound histone acetylation and thereby transcriptional activity [63–66]. More directly, decreased acetylation levels of cccDNA-bound histone H3 can be achieved by histone deacetylase 11 (HDAC11) [67]. In addition, the HBV genome contains three CpG islands as potential substrates for host DNA methyltransferases, whereby methylation of the C residues results in general silencing of transcription [68]. In HBV this reduces pgRNA expression and thus HBV replication [69].

On the virus side, the multifunctional HBx protein has been ascribed to modulate recruitment to cccDNA of epigenetic host factor such as p300, HDAC and SIRT1 [70] and thus to control histone epigenetics [71]. A mechanistically better understood activity of HBx is ubiquitylation and eventually degradation of the structural maintenance of chromosomes 5 and 6 complex (Smc5/6) which, as a restriction factor against episomal DNAs, silences cccDNA transcription [72,73].

HBx counteracts this restriction by usurping, through interaction with DNA damage binding protein 1 (DDB1), a cellular E3 ubiquitin ligase which then ubiquitylates Smc5/6 and tags it for proteasomal degradation. Targeting these protein interactions also opens a wide range for drug design. Nitazoxanid reportedly inhibits the HBx-DDB1 interaction, resulting in inhibition of HBV transcription and viral protein production in human hepatocytes [74]. Pevonedistat is a NEDD8-activating enzyme inhibitor [75]; as NEDDylation is required for E3 ubiquitin ligase activity the inhibitor blocks HBx-mediated Smc5/6 degradation, retaining its restriction factor activity. Better understanding the epigenetic cccDNA regulatory mechanisms will provide further opportunities for the development of novel therapies, and various additional drugs to inhibit transcription are already in development [76•,^77^,^78^].

## Inhibiting the formation of functional HBV capsids

HBV features an icosahedral capsid formed mostly by 120 dimers of the about 180 amino acid long core protein (HBc). Its most obvious role is to provide a stable container for the viral genome yet HBc is dynamically involved in nearly all steps of the viral life cycle [79]. After infection the incoming capsid must disassemble for genome release; for progeny production, newly made HBc must assemble and specifically package pgRNA but no other RNAs or no RNA, probably controlled by HBc phosphorylation [80]. Proper pgRNA reverse transcription occurs only inside the capsid, implying an active role of HBc in the process, again accompanied by changes in phosphorylation status. The capsid is also responsible for proper intracellular trafficking of the viral genome and for providing specific surface interaction sites for envelopment, all at the right time and location, and most, if not all, involving specific host factors. In addition, HBc seems to facilitate viral RNA export from the nucleus and associated with nuclear cccDNA it may be involved in transcriptional regulation. Notably, though, such a function would not depend on *de novo* synthesized HBc [81].

Regarding interference with functional capsid formation, most advanced are small molecules which directly target the core protein and capsid assembly. The first such capsid assembly modulators (CAMs) were AT130, a phenylpropenamide [82], and BAY41-4109, the prototypic heteroaryldihydropyrimidine or HAP molecule [83]. All currently known CAMs bind to the inter-dimer interface of the HBc dimer, allosterically modifying the way how the dimers assemble into the supramolecular capsid structure. Two major modes of action are currently distinguished. HAP compounds generally ‘misdirect’ assembly into aberrant multimers that cannot form closed capsid shells; according to a yet-to-be-approved nomenclature they are classified as CAM-A compounds (A for aberrant). Phenylpropenamides (PPAs) and the more recently identified sulfamoylbenzamides (SBA) derivatives [84] promote formation of seemingly regular but pgRNA-less ‘empty’ capsids; they are hence termed CAM-E compounds (E for empty); however, the actual phenotype can be affected by the extent of binding site occupancy [85]. Hence although the CAMs target the viral core protein, they indirectly have a pronounced impact on HBc’s interaction with other viral factors, for example, polymerase and nucleic acids as well as the envelope proteins, but also with host factors, including kinases and phosphatases, transport proteins, chaperones and others, all of which have to be finely balanced for generation of replication-competent nucleocapsids. Numerous CAMs are currently explored in clinical trials, such as JNJ-56136379, JNJ-6379, ABI-H0731 and NVR 3–778. In a phase 1 study of patients with CHB infection most tested CAMs were well tolerated and demonstrated antiviral activity. Several individuals compounds such as JNJ-56136379 and ABI-H0731 are currently explored in phase II studies [86–89]. The data suggest that the greatest reductions in serum levels of HBV DNA and HBV RNA are achieved in combination with PEG-IFN-α but additional studies are required to better understand the mechanism of action of these molecules.

For instance, unresolved issues are the fate and effects of the misdirected or prematurely assembled HBc multimers which could cause hepatocyte damage and/or even be beneficial for therapy, perhaps by inducing specific immune responses against the cells which harbor them.

## Inhibition of virion assembly and release

HBV produces several types of particles [90], including genome-less, non-infectious types such as the spherical and filamentous subviral particles (SVPs), empty envelopes which account for most of the hepatitis B surface antigen (HBsAg) in serum. However, enveloped rcDNA genome-containing virions are absolutely essential for virus propagation. Their morphogenesis depends on the regulated interaction of rcDNA containing nucleocapsids with the viral envelope proteins, in particular the C terminal part of the preS1 domain and the N terminal part of the preS2 domain (the matrix domain; [91]). The resulting virions are secreted by way of the multivesicular body (MVB) compartment and thus via interaction with numerous cellular factors, many from the sorting complexes required for transport (ESCRT) machinery [92]. Targeting any of these factors should thus reduce formation of infectious virions although as yet no clinical data exist.

More advanced is the inhibition of HBsAg secretion. While not immediately crucial for progeny virus formation as long as enough envelope proteins remain for virion formation the enormous excess of SVPs over virions secreted from HBV infected cells certainly has a benefit for the virus, for example, via a long-assumed decoy activity against virus-neutralizing anti-envelope antibodies and/or by contributing to the inactivation of virus-specific T cell responses during CHB [93]. SVPs are apparently secreted through the classical secretory pathway, not the MVB. Their assembly and secretion can efficiently be inhibited by nucleic acid polymers (NAPs) such as REP-2139 (Figure 1 and Table 1). These modified RNA oligomers possess antiviral activity against several viruses, including HIV-1, HBV and DHBV, both in monotherapy and in combination with immune modulators, and have been shown to reduce or even clear HBsAg from the blood in clinical studies [94,95•,^96^,^97^]. They act in a sequence-nonspecific way and the exact mechanism of action is unclear. Proposedly REP 2139 exerts its activity by interaction with an as yet undiscovered host factor [98]; a recent suggestion is the host Hsp40 chaperone DNAJB12 which is involved in the assembly of SVP (Boulon *et al*., The Hsp40 chaperone DNAJB12 is involved in the morphogenesis of HBV spherical subviral particles and is selectively targeted by nucleic acid polymers. AASLD The Liver Meeting, LP42). Inhibition of HBsAg secretion by NAPs can be reproduced in a human hepatoma cell line which may help to clearly identify the host factor(s) in question [99]. A more detailed understanding of the mechanism-of-action of NAPs may ultimately inform about further optimization of this approach and help in better understanding potential off-target effects.

## Immune modulation

Perhaps the broadest class of HBV-relevant host factors are those from the innate and adaptive immune response and various approaches exploiting immune-mediated antiviral pathways are being pursued [10••] (Figure 1 and Table 1). As summarized in [4] toll-like receptor (TLR) agonists, therapeutic vaccines, immune checkpoint inhibitors and engineered T cells are currently investigated as therapeutic strategies for CHB treatment since several comprehensive reviews on this topic are available [100–105].

We here focus on only a few select aspects. Activation of TLR3, TLR7/8 and TLR9 can reduce HBV replication *in vitro* and *in vivo* through production of antiviral cytokines such as IFNs and activation of natural killer (NK) and T cells [106–108]. Several TLR agonists such as RO7020531, JNJ-4964, GS-9620 and GS-9688 are being explored for the treatment of CHB in clinical trials [109]. Notably, an antiviral effect of TLR7 agonist GS-9620 (vesatolimod) was clearly demonstrated in chronically woodchuck hepatitis B virus (WHV) infected woodchucks and in HBV infected chimpanzees, with decreases in cccDNA and surface protein levels (WHsAg or HBsAg) [107,110]. However, in a clinical phase 2 study on safety and efficacy jn viremic CHB patients vesatolimod had no significant anti-viral activity [111]. If this also holds for other TLR agonists their main potential may be to increase anti-HBV immune responses in combination therapies. A RIG-I agonist, inarigivir (or SB 9200) also decreased viral DNA and RNA in CHB patients [109]. Not the least, various strategies to improve HBV-specific T cell responses in CHB patients are being explored [101,102]. Several therapeutic vaccines are in clinical phase 1 [103], all aiming to overcome shortcomings of classical vaccines by advanced prime-boost immunization schemes, for example, by combining virally vectored plus adjuvanted proteins as immunogens, as in GSK3528869A (clinical trial identifier NCT03866187 at ClinicalTrials.gov). Another actively pursued principle is the use of immune checkpoint inhibitors, that is, anti-programmed cell death-1(PD-1), anti-PD-Ligand1 (anti-PD-L1) and anti-CTLA-4 (clinical phase 1) [100]. These approaches show great promise in cancer therapy by blocking inhibitory signaling on CD8+ T cells by cancer cells [112], and HBV-infected hepatocytes may employ similar immune resistance mechanisms. Recent data indicate, however, that HBV-specific CD8+ T cells primed by hepatocytes cannot be activated by PD-L1 blockade. Yet, they do respond to interleukin 2 (IL-2) [113] which may hence be exploited as a basis for alternative immunotherapy strategies.

## Conclusions

The finding that patients can spontaneously eliminate HBV infection suggests that the development of curative therapies is an achievable goal. Recent knowledge in virus–host interactions from studies in cell-based and animal model systems has uncovered novel options to exploit host-dependency factors and the innate immune system to target the numerous steps in the HBV life cycle. Many host dependency factors targeting approaches are still in the discovery or preclinical stage, but several immune targeting therapies have already reached clinical development.

The increased barrier to pathogen resistance development upon targeting host factors, and the proneness to side-effects of host factor inhibition are two sides of the same medal. It is therefore difficult to predict which host factor targets are the most promising and under which circumstances. Clearly, limiting the duration and localization of host factor inhibition would help to minimize side effects but for most of the host factor targets outlined above much more data are required for strong judgements. Reassuringly, though, one of the host factors discussed above, NTCP, already provides clinical proof that the benefit of antiviral activity can outweigh minor side effects [19••,^20^]. Virtually all patients treated with Bulevirtide experienced moderate increases in serum bile acid levels, as expected from blocking a bile acid transporter. However, this increase was well tolerated by all study participants up to 48 weeks of treatment and so was no concern for the European authorities in the approval of the compound as first HDV treatment [49••]. Results of Bulevirtide monotherapy were less convincing regarding CHB but this may relate to the fact that HDV as an RNA virus lacks a stable persistence reservoir such as HBV cccDNA and therefore is more dependent on ongoing genome replication.

However, in combination regimens Bulevirtide also showed some promise regarding CHB treatment (see below). Of the other host factors outlined above as potential HBV targets those involved in cccDNA biogenesis might be closest to the sterilizing cure goal. This view is supported the positive impact of entry blockade on viral titers which implies that HBV persistence relies not only on cells stably harboring, or intracellularly recycling, cccDNA but on infection of naive hepatocytes. If cccDNA did not undergo turnover and cccDNA harboring hepatocytes lived forever, blocking rcDNA to cccDNA conversion would be fruitless. If, however, the pool of cccDNA is constantly replenished by infection, then inhibiting cccDNA neogenesis can be expected to have therapeutic benefit.

Most of the host factors identified as important for rcDNA to cccDNA conversion, especially the five factor core set [34•], are essential for cellular DNA replication. Hence it could be hoped that certain cancer treatments that rely on targeting rapidly dividing cells might also be applicable to prevent/suppress cccDNA formation without too much impact on the usually slowly (once in a few months) dividing hepatocytes. However, here as with other DNA replication and DNA repair factors more data are needed to correlate how long an inhibition can be tolerated by the host but significantly impair HBV persistence.

Generally, however, while the efficacy of monotherapies may be high, as for NUCs, it is still insufficient to warrant complete viral suppression. Hence combination therapies appear to have great potential. One reason is that while it is biologically feasible to achieve 99% inhibition, reaching 100% is very difficult if not impossible. In combination therapy this is accounted for by ‘synergy’ of two or more components, that is, that the whole is greater than the sum of the individual parts [114]. An example is that combination, in particular when targeting host factors, allows to reduce the dose of an inhibitor to a subtoxic level while maintaining, by synergy with the combination partner, therapeutic efficacy.

Indeed, several clinical studies suggest such an added benefit. Earlier studies concluded that combining NUCs, including ETV [115], with type-I IFN had no extra benefit but a newer study [116] combining TDF plus PEG-IFN led to enhanced HBsAg seroclearance (though perhaps with some HBV genotype bias and still in less than 10% of patients); the seemingly discrepant data might also indicate that minor differences between two potent antivirals such as TDF and ETV can eventually tip the balance towards a more efficient immune response. Also combining bulevirtide with PEG-IFNa-2a substantially increased the fraction of patients experiencing HBsAg seroclearance after 48 weeks of treatment over either drug alone. Hence weakening the virus appears to strengthen the antiviral potency of the immune system. While these data are promising a severe limitation of IFN-involving combinations is the non-eligibility of many CHB patients for IFN therapies, owing to its severe adverse effects. Perhaps these can be alleviated by small molecule IFN inducers such as the TLR or RIG-I agonists. In general, however, exploiting components, including engineered ones, of the adaptive immune system as one arm of combination therapies may be a more versatile approach.

The overall goal will be to maximize interference with viral multiplication, prevent viral escape, and help the host immune system to regain control. As different modes of action may be most advantageous combining an effective direct antiviral agent [87] with a host-factor targeting agent plus an immune stimulus appears quite promising. Some clinical data are already available and they clearly show an added benefit of adding the host-factor targeting NAP to a combination of NUC plus PEG-IFN [117]. As we are approaching a post-IFN era two or more non-immune drugs may already achieve a sufficient antiviral efficacy to enable the endogenous immune system by itself to regain control. This is a rationale for cooperative phase 2 clinical trial by Assembly Bio and Arbutus of a triple combination therapy including a NUC, a CAM, and a HBsAg reducing siRNA (ClinicalTrials.gov Identifier: NCT04820686).

In the light of the increasing number of targets for each class of antiviral we are likely to see many more double and triple combination trials in the near future. While this increases the number of options to test it also boosts chances that one or more of these approaches will bring us closer to a cure of CHB, likely with a significant contribution from host factor targeting agents.

Given the complexity of the HBV life cycle, it is likely that only such combination therapies, for example, the integrated use of direct-acting antiviral(s) and immune-activating approaches, will be able to achieve the eventual goal of cccDNA elimination. Hence further studies are needed to understand and assess the efficacy and safety of the therapeutic strategies that are already in clinical trials and develop advanced treatment modalities [4,87,118]; a more detailed elucidation of virus–host interactions, in particular cccDNA biology and the mechanisms of cccDNA formation and elimination, will add clues to advance new, innovative concepts into the pre-clinical and clinical trial stage.

## Figures and Tables

**Table 1:** Examples for host-targeting approaches for HBV treatment in preclinical and clinical development and approved

Coumpound	Targets	Concept	Stage of development	References
Bulevirtide	Entry host factors NTCP	Prevention of binding and entry into the cells	Clinical studies	[46–48,49••]
Cyclosporin A			Discovery/preclinical	[42–45]
Interleukin-6	Decrease cccDNA-bound histone acetylation			[63–66]
Histone deacetylase 11				[67]
DNA methyltransferase	Methyl group to CpG islands	Silencing of cccDNA transcription	Discovery/preclinical	[68,69]
Nitazoxanid	Inhibits HBx-DDB1 interaction			[74]
Pevonedistat	NEDD8-activating enzyme inhibitor			[75]
Nucleic acid polymers (REP-2139)	Hsp40 chaperone DNAJB12?	Inhibiting HBsAg assembly and release	Clinical studies	[94,95•,^96^–^98^]
RO7020531	^a^TLR agonist	Cytokine production	Clinical studies	[106–111]
JNJ-4964				
GS-9620				
GS-9688				
PEG-interferon α	Decrease cccDNA-bound histone acetylation (amongst others)	Silencing of cccDNA transcription	Approved	[4]

a TLR: toll-like receptor.
